# “Simple Rules for Editors”? Here Is One Rule to Tackle Neglected Problems of Publishing: Response from PLoS

**DOI:** 10.1371/journal.pcbi.0030089

**Published:** 2007-05-25

**Authors:** Mark Patterson, Barbara Cohen

We are grateful to the Drs. Erren for highlighting the concern that younger and less-experienced scientists are sometimes not given due credit for their contributions, in particular through omission from authorship on published articles. The Drs. Erren correctly point out that many journals including those published by PLoS do ask for all author contributions to be described on submission, and these contributions are also included in the published article. The corresponding author has ultimate responsibility for ensuring that these contributions are correct.

Authorship issues are a topic of considerable debate among editorial groups such as the International Committee of Medical Journal Editors (ICMJE) and the Committee on Publication Ethics (COPE). *PLoS Medicine,* for example, requires that each author on a paper respond to a specific e-mail to independently confirm their contribution to the work and whether they have any competing interests. No final decision is made on the paper until all authors have responded to that e-mail. This policy aims to ensure that all listed authors are able to justify their inclusion and to doublecheck for competing interests. Although it is not the role of editors to arbitrate authorship, on two occasions authors have agreed after receiving this e-mail request that they do not fulfil the criteria for authorship and have requested that they simply be acknowledged within the paper instead. In addition, *PLoS Medicine* specifically reminds authors on submission that all medical writers must be included on the paper with their contributions.

PLoS is committed to ensuring that the byline on papers is correct and that the contribution statement accurately describes the contributions of authors. One way to reinforce to authors the importance of accurate authorship details is to strengthen our statement on submission forms and on author instructions for all journals, and to remind corresponding authors that they are ultimately responsible for confirming that no additional authors should be listed and that author contributions are accurate. It will also be interesting to see whether issues relating to authorship will be addressed with the commentary and annotation features that are available in the new journal *PLoS ONE,* and which will be applied to other PLoS journals in due course.

